# Retrospective Evaluation of Acute Kidney Injury and Its Association With Severity and Outcome in Small Animal Trauma Patients: 387 Cases (2017–2021)

**DOI:** 10.1111/vec.70006

**Published:** 2025-07-29

**Authors:** Emily Jane Stacey, Stefano Cortellini, Laura Pearl Cole

**Affiliations:** ^1^ Department of Clinical Science and Services, Section of Emergency and Critical Care, The Royal Veterinary College University of London North Mymms Hertfordshire UK

**Keywords:** acute kidney injury, Animal Trauma Triage score, fluid responsive, hospital‐acquired AKI, trauma

## Abstract

**Objective:**

To describe the prevalence of acute kidney injury (AKI) in canine and feline trauma and to determine associations between AKI and trauma severity, outcome, species, and other factors.

**Design:**

Analysis of cases submitted by one hospital to the Veterinary Committee on Trauma registry between April 2017 and February 2021 that had blood creatinine concentration measured within 6 h of presentation after trauma.

**Setting:**

University teaching hospital in the United Kingdom.

**Animals:**

A total of 220 canine and 167 feline trauma patients.

**Measurements and Main Results:**

Azotemic AKI was defined as creatinine concentration > 140 µmol/L (1.58 mg/dL) and subgrouped by fluid responsiveness according to the International Renal Interest Society. Hospital‐acquired AKI (HAAKI) was defined as a serial increase in creatinine concentration ≥ 26.4 µmol/L (≥ 0.3 mg/dL) from a nonazotemic baseline. Trauma severity, Animal Trauma Triage (ATT) score, survival, age, and species were compared between groups with and without AKI. Twenty‐eight of 387 (7.24%) cases (23/167 [13.8% cats]; 5/220 [2.3% dogs]) had AKI on presentation. Cats were more likely to present with AKI than dogs (odds ratio: 4.95; 95% confidence interval, 2.36–10.8; *P* < 0.0001). Nine of 17 (52.9%) azotemic AKI patients in which serial creatinine concentrations were available had fluid‐responsive AKI. HAAKI was documented in seven of 105 patients (6.67%). Median ATT score on presentation was higher in azotemic AKI than non‐AKI cases (*P* = 0.02). Twenty‐two of 28 (78.6%) azotemic AKI cases, three of seven (42.9%) HAAKI cases, and 316 of 359 (89.8%) non‐AKI cases survived.

**Conclusions:**

AKI occurs in canine and feline trauma and appears associated with higher trauma severity. Its impact on survival requires further investigation.

**Abbreviations**
AKI: acute kidney injury
ATT: Animal Trauma Triage score
CKD: chronic kidney disease
HAAKI: hospital‐acquired acute kidney injury
IQR: interquartile range
IRIS: International Renal Interest Society
ISS: Injury Severity Score
non‐AKI: nonacute kidney injury
OR: odds ratio
REDCap: Research Electronic Data Capture
UOP: urine output

## 1 Introduction

Acute kidney injury (AKI) is a spectrum of injuries to the kidneys that can lead to an acute decrease in kidney function. In people, AKI is a common sequela to trauma, with incidences ranging from 12.4% to 25%, and has been associated with increased mortality [1, 2].

Trauma accounts for a large proportion of small animal veterinary practice cases; however, evidence regarding the incidence of AKI in this population is limited [3, 4, 5]. In one publication describing cats with AKI, four of 45 (8.89%) had a history of trauma [6]. In dogs, one case report demonstrates AKI secondary to traumatic rhabdomyolysis [7]. The literature regarding hospital‐acquired AKI (HAAKI) in small animals is also limited. One study reported 62 of 97 (64%) canine ICU patients developed AKI International Renal Interest Society (IRIS) Grade I and another study showed 24 of 164 (14.6%) canine ICU patients developed an AKI based on the novel Veterinary AKI staging system [8, 9]. Higher veterinary ICU mortality has been associated with increasing serum creatinine concentration over the course of the hospitalization when compared with those patients presenting with increased creatinine levels that decreased over time [9].

Risk factors for AKI in human trauma include male sex, increasing age, massive transfusion, prehospital mean arterial blood pressure value, maximum prehospital heart rate, secondary transfer to a trauma center, renal trauma, blood lactate concentration, dysglycemia, hemorrhagic shock, and peak creatine kinase concentration [1, 10, 11, 12]. In people, a higher Injury Severity Score (ISS) is associated with an increased risk of AKI [1, 2, 11] The Animal Trauma Triage (ATT) score is the validated veterinary equivalent of these scales and was not associated with AKI in a feline study on multiple organ dysfunction syndrome [13, 14].

The primary aim of the current study was to describe the prevalence, grades, and onset of AKI in canine and feline trauma patients. Second, we wanted to identify whether AKI was associated with trauma severity, hospitalization length, and outcome. In addition, we wanted to describe the frequency of previously reported risk factors for AKI in this specific population.

## 2 Materials and Methods

A search was performed of the Veterinary Committee on Trauma Research Electronic Data Capture (REDCap) database (grant number UL1TR002494, National Institutes of Health's National Centre for Advancing Translational Sciences) to identify emergency primary care and referred trauma cases submitted to the database specifically by our hospital between April 2017 and February 2021. Patients were included if they had a trauma recorded and at least one blood creatinine concentration measured within 6 h of presentation to our hospital. Patients were excluded if initial blood tests were performed > 6 h after presentation, if data were incomplete (e.g., no blood sampling, machine error, or records unavailable), or if the cause of AKI was classified exclusively as “postrenal” (e.g., diagnosis of uroabdomen, urethral rupture, urinary bladder herniation), because in such cases the pathophysiologic mechanism of azotemia is typically different from that of intrinsic or fluid‐responsive AKI. Patient data were recorded, including signalment, type of trauma (blunt, penetrating, mixed), creatinine concentrations (presentation and serial measurements during hospitalization, if available), ATT score as recorded in the REDCap database, urinalysis (including sediment analysis when available), urine output (UOP), admission type (referral vs. primary accession), other admission variables (blood glucose and lactate concentrations, where available), treatment in hospital (e.g., general anesthesia, blood transfusion, mechanical ventilation requirement, when available), length of hospitalization (of survivors), and survival to discharge. For nonsurvivors, death or the discernible reason for euthanasia was recorded.

Serum biochemistry analysis was performed on a point‐of‐care or laboratory analyzer.^a^^,^ b Azotemia was defined as creatinine concentration ≥ 140 µmol/L (1.58 mg/dL) [15]. Azotemic AKI on presentation was defined as blood or serum creatinine concentration ≥ 140 µmol/L without evidence of chronic kidney disease (CKD) in the available medical history (e.g., history or physical examination, previous biochemistry panels, or imaging findings suggestive of CKD). HAAKI was defined as patients that were nonazotemic on presentation and had a documented increase in blood creatinine concentration ≥ 26.4 µmol/L (≥ 0.3 mg/dL) during hospitalization [16]. AKI was graded based on IRIS guidelines and was further categorized as oliguric (< 1 mL/kg/h), anuric (0 mL/kg/h over 6 h), or fluid‐responsive AKI [15]; evidence of kidney injury on urinalysis (proteinuria with inactive sediment or glucosuria with normoglycemia, presence of urinary casts); or imaging findings suggestive of AKI [15]. Fluid‐responsive AKI was defined as the return of blood creatinine concentration to < 140 µmol/L within 48 h of fluid therapy [15]. Postrenal AKI was considered to be due to an obstructive process distal to the kidney (e.g., ureteric injury, uroabdomen). A mixed AKI was considered if an azotemia persisted despite management of identified prerenal and postrenal causes of azotemia. Hospital records and those from the referring practice (where applicable) were reviewed to identify potential risk factors for AKI, including nonsteroidal administration, nephrotoxic antimicrobials, general anesthetic or sedation, surgical procedure, and the use of contrast medium.

Descriptive statistics were performed using a commercial statistical application.^c^ Data were assessed for normality using the Shapiro–Wilks test and visual inspection of histograms. Continuous data were reported as median (interquartile range [IQR]) or mean (±SD) according to distribution. The Fisher's exact test was used to compare two categorical groups, while the Mann–Whitney *U*‐test compared continuous variables across non‐AKI and azotemic AKI groups. To assess for any differences in AKI prevalence between dogs and cats and between sexes, the odds ratio (OR) was calculated using the Baptista–Pike method if significance was achieved [4]. Statistical significance was set at *P* < 0.05.

## 3 Results

**FIGURE 1:**
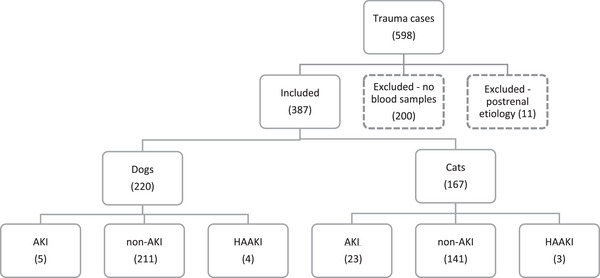
Distribution of 220 dogs and 167 cats presenting to a university hospital in the United Kingdom with trauma, with case numbers listed by species and by AKI, non‐AKI, and HAAKI. AKI, acute kidney injury; HAAKI, hospital‐acquired AKI; non‐AKI, no AKI.

Five hundred ninety‐eight cases (348 dogs, 250 cats) presented with trauma during the study period; 200 cases were excluded due to incomplete data or postrenal azotemia (11), leaving 387 animals in the final analysis (220 dogs, 167 cats; Figure 1). The median time to presentation from trauma, where this was recorded, was 4.5 h (IQR: 1.5–8.25). Median creatinine concentration on presentation was 64 µmol/L (IQR: 46–92 µmol/L [0.72 mg/dL]; IQR: 0.52–1.04 mg/dL; Table 1). Canine and feline signalment, injury type, and outcome type are summarized in Table 2.

**TABLE 1:** Median blood creatinine concentration at presentation for 220 dogs and 167 cats presented for trauma.

	Median blood creatinine concentration at presentation	Trauma population	AKI	Non‐AKI	HAAKI
Total	µmol/L (IQR) mg/dL (IQR)	(*n* = 387) 64 (46–92) 0.74 (0.52–1.0)	(Total *n* = 28) 164 (153–234) 1.9 (1.7–2.6)	(*n* = 352) 62 (44–85) 0.70 (0.50–0.96)	(*n* = 7) 113 (45–128) 1.3 (0.51–1.45)
Feline		(*n* = 167) 52 (37–67) 0.59 (0.42–0.76)	(*n* = 23) 159 (153–209) 1.8 (1.7–2.4)	(*n* = 141) 84 (67–100) 0.95 (0.76–1.1)	(*n* = 3) 104 (74–128) 1.2 (0.8–1.4)
Canine		(*n* = 220) 51 (37–66) 0.58 (0.42–0.75)	(*n* = 5) 253 (155–764) 2.9 (1.8–8.6)	(*n* = 211) 51 (36–64) 0.58 (0.41–0.72)	(*n* = 4) 116 (62–125) 1.3 (0.70–1.4)

Abbreviations: AKI, acute kidney injury; HAAKI, hospital‐acquired AKI; IQR, interquartile range; non‐AKI, no AKI.

Total study population, patients with AKI, those without AKI, and those with HAAKI are presented together and divided by species.

**TABLE 2:** Dogs and cats presenting for trauma that had blood creatinine concentration measured within 6 h of presentation.

	Inclusion	Azotemic AKI	Non‐AKI	HAAKI
Number	**387**	**28/387**	**352/387**	7/105*
Patient source				
Primary accession	82	10	72	0
Referral	305	18	280	7
Cats	**167**	**23**	**141**	**3**
Pedigree	39	5	32	1
DSH, DMH, DLH	119, 1, 8	18	102, 1, 6	2, 0, 0
Breed of dog	**220**	**5**	**211**	**4**
Pedigree	175	5	168	3
Mixed breed	45	0	43	1
Dog weight	**220**	**5**	**211**	**4**
Small: < 5 kg	50	2	48	0
Medium: 5–19.9 kg	121	2	116	3
Large: 20–39.9 kg	49	1	47	1
Giant: > 40 kg	0	0	0	0
Median age (IQR), years				
Dogs	2.7 (0.7–6)	3 (1.4–10.1)	2.5 (0.6–6.0)	7 (5.5–13.2)
Cats	3.3 (1.5–6.1)	4.6 (1.9–9.9)	3.1 (1.2–6.0)	5.8 (4–12.3)
Dogs				
Male	**121**	**5**	**115**	**1**
Neutered	58	3	54	1
Intact	63	2	61	0
Female	**99**	**0**	**96**	**3**
Neutered	51	0	49	2
Intact	48	0	47	1
Cats				
Male	**89**	**13**	**76**	**0**
Neutered	74	13	61	0
Intact	15	0	15	0
Female	**78**	**10**	**65**	**2**
Neutered	63	8	52	2
Intact	15	2	13	0
Type of trauma				
Blunt	291 (75.2%)	21 (75%)	267 (75.6%)	3 (42.9%)
Penetrating	65 (16.8%)	4 (14.3%)	60 (17%)	1 (14.3%)
Mixed	31 (8%)	3 (10.7%)	25 (7.1%)	3 (42.9%)
Outcomes				
Died naturally	14 (3.62%)	2 (7.14%)	9 (2.56%)	3 (42.9%)
Euthanized	**27 (6.98%)**	**4 (14.3%)**	**22 (6.25%)**	**1 (14.3%)**
Grave prognosis	22	3	19	0
Prognosis + finances	3	1	1	1
Financial limitations	2	0	2	0
Survived	341 (89.9%)	22 (78.6%)	316 (89.8%)	3 (42.9%)
Unknown outcome	5 (1.29%)	0	5 (1.42%)	0

*Note*: Of the 352 non‐AKI cases, 105 had serial blood samples (*) taken while in hospital, which identified seven cases with serial creatinine increases consistent with AKI. Outcome was reported as unknown if there were no clear data in hospital records. In cells with subsets of animals represented, **bolded** values represent the total number of individuals in the set while plain font values represent subsets of that bolded total.

Abbreviations: AKI, acute kidney injury at presentation; DLH, domestic longhair; DMH, domestic medium‐hair; DSH, domestic shorthair; HAAKI, hospital‐acquired AKI; IQR, interquartile range; non‐AKI, no AKI.

Animals are grouped as those with azotemic AKI on presentation, non‐AKI on presentation, and HAAKI.

### 3.1 Prevalence of Azotemic AKI

Twenty‐eight of 387 (7.24%) small animal trauma patients had azotemic AKI on presentation, including 23 of 167 cats (13.8%) and five of 220 dogs (2.27%). Cats were more likely to present with azotemic AKI than dogs (OR: 4.95; relative risk: 4.3; 95% CI, 2.36–10.8; *P* < 0.0001). Grade II AKI was the most common grade of azotemic AKI cases (19/28 [67.9%]); two cases had Grade V AKI (Table 3). Serial blood results were available for 17 of the 28 cases that presented with AKI and for 105 of 359 cases that were nonazotemic on presentation. UOP data were available for one case (anuric), and urine samples were available in four cases, with examination limited to a dipstick examination (proteinuria in two cases, glucosuria in one case, and no abnormalities in the fourth case).

**TABLE 3:** International Renal Interest Society (IRIS) grade of patients with azotemic acute kidney injury on presentation based on species (canine or feline) and survival characteristics.

IRIS grade	Canine (*n* = 5) %	Feline (*n* = 23) %	No. of nonsurvivors		No. of survivors—(%)
			Died	Euthanized	
Grade I	0	0	0	0	N.A.
Grade II	2 (40%)	17 (73.9%)	0	2	17/19 (89.5%)
Grade III	2 (40%)	5 (21.7%)	1	2	4/7 (57.1%)
Grade IV	0	0	0	0	N.A.
Grade V	1 (10%)	1 (4.3%)	1	0	1/2 (50%)

Reasons for nonsurvival categorized as died or euthanized. Values reported as overall case numbers with percentages written in brackets.

The type of trauma sustained was similar between species and groups (trauma population, azotemic AKI and non‐AKI), with the majority experiencing blunt trauma (291/387 [75.2%]; Table 2).

### 3.2 Azotemic AKI Subcategorization

Of the 28 cases with azotemic AKI at presentation, 17 had serial creatinine measurements, nine (52.9%) of which were classified as fluid responsive. Two of 28 (7.14%) cases had a postrenal component and persistence of azotemia despite surgical resolution of obstruction; therefore, these cases were considered mixed. The 11 cases without serial creatinine measurements could not be classified further. One case was anuric, and no cases were reported to be oliguric.

### 3.3 Hospital‐Acquired AKI

Serial creatinine concentration measurements were available for 105 of 359 (29.2%, 52 dogs, 53 cats) nonazotemic animals on presentation, of which seven (6.67%) experienced an increase in creatinine concentration ≥ 26.4 µmol/L (≥ 0.3 mg/dL), compatible with Grade I and II AKI (Table 1). In four of seven cases, this increase occurred within 48 h, whereas two cases’ creatinine concentrations increased at 72 h and in one case at 216 h (all Grade II).

Median creatinine concentration at 48 h was approximately 145.5 µmol/L (IQR: 88–194 µmol/L [1.6 mg/dL; IQR: 0.99–2.2 mg/dL]). The median time between presentation and AKI developing was 19.5 h (IQR: 2–36 h). UOP was unknown for the cases with HAAKI cases and urinalysis was not performed for any of these cases.

### 3.4 Animal Trauma Triage Score

Median ATT score in AKI dogs was 4 (IQR: 1–6.5) and in cats was 3 (IQR: 2–6). When the two species were combined, ATT score was significantly higher in AKI cases (3; IQR: 2–6) than in non‐AKI cases (2; IQR: 1–4; *p* = 0.01). Median ATT score for patients with HAAKI was 5 (IQR: 2–5); for cats, the score was 5 (IQR: 1–5), and for dogs, the ATT score was also 5 (IQR: 2.75–5.75).

### 3.5 Outcomes: Length of Hospitalization

Of patients that survived to discharge, the median hospitalization length was 3.5 days (IQR: 1.3–5.8) for azotemic AKI cases and 4 days (IQR: 2–6) for non‐AKI cases (*P* = 0.054). For HAAKI cases, hospitalization length was 5 days (IQR: 4–13).

### 3.6 Outcomes: Survival to Discharge

Overall, 316 of 352 (89.8%) non‐AKI animals survived to discharge (123/142 cats [86.6%] and 193/210 dogs [91.9%]). Twenty‐two of 28 (78.6%) animals presenting with azotemic AKI survived to discharge (18/23 [78.3%] cats; 4/5 [80%] dogs). There was no difference in survival comparing AKI and non‐AKI patients (*p* = 0.09). There was no significant difference between creatinine concentrations of survivors versus nonsurvivors within the AKI category (*P* = 0.08). Of the six animals with AKI at presentation that did not survive to discharge, four were euthanized and two died (Table 3). Of the 31 cases without AKI for which details of nonsurvival to discharge were known, 19 were euthanized for grave prognosis, nine died, two were euthanized for financial reasons, and one was euthanized for a combination of poor prognosis and financial concerns. Three of seven (42.9%) HAAKI cases survived to discharge; of those that did not survive, three died and one was euthanized due a combination of grave prognosis and financial limitations (Table 2).

### 3.7 Other Admission Variables

The median blood glucose concentration on presentation in the azotemic AKI group was 8.3 mmol/L (IQR: 6.4–12.4 mmol/L [150 mg/dL]; IQR: 115–224 mg/dL, *n* = 22), which was significantly higher than the admission blood glucose concentration in the non‐AKI group of 6.7 mmol/L (IQR: 5.9–8.7 mmol/L [121 mg/dL]; IQR: 106–156 mg/dL, *n* = 320; *P* = 0.02). The median lactate concentration on presentation in the azotemic AKI group was 2.1 mmol/L (IQR: 1.3–3.9 mmol/L [37.8 mg/dL]; IQR: 23.4–70.2 mg/dL, *n* = 24) and in the non‐AKI group was 1.6 mmol/L (IQR: 1.2–2.2 mmol/L [29 mg/dL]; IQR: 21.6–39.6 mg/dL, *n* = 315; *P* = 0.003). For cases presenting with HAAKI, the blood glucose concentration was 7.6 mmol/L (IQR: 5–11.8 mmol/L [137 mg/dL]; IQR: 90–212 mg/dL), and lactate concentration was 2.1 mmol/L (IQR: 1–6.67 mmol/L [37.8 mg/dL]; IQR: 28.8–120 mg/dL, *n* = 5).

The median age of dogs and cats in the study was 2.7 years (IQR: 0.7–6.0 years) and 3.3 years (IQR: 1.5–6.1 years), respectively. Of dogs that presented with AKI, the median age was 3 years (IQR: 1.4–10.1 years), for non‐AKI cases, the median age was 2.5 years (IQR: 0.6–6.0), and the median age was 7 years (IQR: 5.5–13.3 years) for HAAKI cases. The median age of cats presenting with AKI was 4.6 years (IQR: 1.9–9.9 years), for non‐AKI cases was 3.1 years (IQR: 1.2–6.0), and for cats with HAAKI, 5.8 years (IQR: 4–12.3 years). There was no difference in age between cats that presented with AKI versus non‐AKI (*P* = 0.08), or in age between dogs that presented with AKI versus non‐AKI (*P* = 0.67). There was no association between sex and presentation with AKI versus non‐AKI (*P* = 0.32).

### 3.8 Medications and Procedures in AKI Cases

Nephrotoxic drug administration was documented before presentation in seven of 28 cases presenting with azotemic AKI, three of which were deemed to have fluid‐responsive AKI. Meloxicam was administered in six, furosemide in three cases, and no cases received contrast medium before referral. Of the seven HAAKI cases, one received meloxicam and one was sedated before referral. Of those cases that developed HAAKI, four of seven had computed tomography with contrast under sedation or general anesthetic during hospitalization, with three of these cases being documented after computed tomography with contrast medium administration. A surgical procedure was required in 18 of 28 (64.3%) azotemic AKI cases, 250 of 352 (71%) non‐AKI cases, and five of seven (71.4%) HAAKI cases. Overall, 13 of 352 (3.7%) non‐AKI cases, two of 28 (7.1%) azotemic AKI cases, and two of seven (28.6%) HAAKI cases required a blood transfusion during hospitalization. Mechanical ventilation was performed in one of 28 (3.6%) azotemic AKI case and in zero of 359 non‐AKI cases.

## 4 Discussion

The prevalence of azotemic AKI on presentation in this trauma population was 7.24% (13.8% in cats and 2.3% in dogs). A recent feline study of polytrauma cases [14] found a prevalence of 32%, which is higher than our study. A potential explanation for this discrepancy is that in the aforementioned study, only cats that required ICU admission were included, suggesting a more severe trauma population, and prospective recruitment facilitated serial creatinine concentration measurement alongside UOP to better capture early AKI cases. One human study reported that approximately a quarter of trauma patients develop AKI [17], which is higher than the current study for reasons such as scoring systems that focus on relative change of creatinine compared with baseline and availability of UOP data; therefore, IRIS Grade I equivalent cases are less likely to have been missed.

IRIS Grade II AKI was most common in dogs and cats with AKI (40% and 73.9%, respectively), which is comparable to human literature where lower Kidney Disease Improving Global Outcomes stages and Risk of renal dysfunction, Injury to kidney, Failure or Loss of kidney function, and End‐stage kidney disease (“RIFLE”) scores are more common in the trauma population [10, 17, 18]. In the current study, Grade V AKI was documented in one dog and one cat (7% AKIs), lower than the human literature, where 28% had Kidney Disease Improving Global Outcomes Stage 3 (which could be compared loosely to IRIS Grade III–IV+) [10]. Veterinary patients may be less likely to have a higher AKI grade due to the impact of owner decision‐making (i.e., euthanasia) in cases of severe polytrauma, making identification of AKI less likely.

In the cases in which serial blood work was available, more than 50% of animals with AKI were classified as having fluid‐responsive AKI, which is higher than previously reported in dogs with all‐cause AKI. This is perhaps because of the higher likelihood of hypovolemia in a trauma patient [19]. The data likely underestimated volume‐responsive AKI due to the patient population largely being a referral population, often receiving fluids before presentation and with creatinine concentrations being measured a variable amount of time after trauma. The retrospective nature of the study meant a reduced availability of serial creatinine concentration and UOP measurements. Azotemia persisted in some cases of AKI, suggesting intrinsic injury; however, incomplete serial data mean the true prevalence cannot be determined. Postulated mechanisms for an intrinsic AKI in trauma include oxidative stress, inflammation, epithelial dysfunction, direct kidney injury, nephrotoxic therapies, abdominal compartment syndrome, and rhabdomyolysis [1, 2, 20]. Further studies could assess creatine kinase concentrations as these data were not available in the current study.

Seven of 105 (6.67%) patients with serial creatinine concentrations that were nonazotemic on presentation developed HAAKI. This finding is in line with recent retrospective studies of HAAKI in dogs, whereby 9%–14.6% of dogs presenting with a variety of conditions for nonroutine admission, including hospitalization in the ICU, were diagnosed with HAAKI [9, 21, 22]. However, this finding is markedly lower than a study of hospitalized dogs that showed 64% developed an IRIS Grade I AKI. The results of our study also show a lower prevalence compared with human trauma patients, in which HAAKI is reported in 12.4%–25% of cases [1, 10, 17]. Some of this difference may be related to the use of different AKI grading systems [23]. The majority of HAAKI cases (6/7 [85.7%]) in the current study were documented within 72 h of presentation, which is concordant with human literature suggesting 96% of AKIs occur within 5 days of trauma [11]. Given the majority of this population was referred to our hospital, it is possible that many HAAKI cases were misclassified as AKI cases because they were azotemic at the time of arrival to our hospital. Furthermore, lack of serial blood work in the nonazotemic population may have resulted in cases being missed; thus, the true incidence of HAAKI in dogs and cats with trauma remains unknown. Further studies are needed to investigate the prevalence and risk factors for HAAKI in veterinary trauma patients.

The current study found cats (OR: 4.95; 95% CI, 2.36–10.8; *p* < 0.0001) were more likely to present with AKI than dogs (OR: 0.20; 95% CI, 0.09–0.42). Although not previously assessed, this corresponds with the feline literature, where trauma is more frequently cited as a cause of AKI in cats than dogs [6, 7, 14, 24, 25]. It could also be explained by the relatively small size of cats compared with dogs, meaning that trauma may cause more severe injury, as per the differences seen in predicted mortality between adult and pediatric patients using the ISS [26]. However, it is important to note the higher physiological reserve in pediatric patients compared with adults, meaning that factors other than size may contribute to this difference [26]. Second, this may reflect a difference in the species and AKI classification, as current IRIS guidelines do not differentiate species and the distinct reference intervals of various machines.

Another explanation for cats being more likely to present with AKI than dogs could be hypotension; a study evaluating ATT score in cats after a dog bite showed a higher score to be associated with a lower blood pressure, and other studies document ischemia/reperfusion injury as a mechanism of veterinary AKI after trauma [27, 28]. Although blood pressure was not assessed in the current study, our finding that lactate concentration was significantly higher in AKI cases than non‐AKI cases (*p* = 0.003) is comparable with human literature and may indicate that more severe hypoperfusion may have led to renal ischemia [1, 11, 17]. A study found 35.1% of cats to be in circulatory shock when presenting to primary care veterinarians after a trauma, perhaps indicating their susceptibility to AKI via reduced renal perfusion and other mechanisms [24, 29, 30]. Furthermore, given reports of a higher prevalence of kidney disease in cats overall, and based on the retrospective nature of the study, it may be that cases of preexisting CKD were present and not accurately excluded when reviewing medical histories [31]. Other mechanisms of AKI, including inflammation, oxidative stress, and epithelial dysfunction, may have different roles across species [28].

Trauma severity as determined by ATT score was significantly greater in AKI cases than in non‐AKI cases, and while there were too few HAAKI cases to perform a statistical analysis, median ATT score for these cases was higher than for non‐AKI and AKI cases. This result is comparable to human studies, in which a high ISS is considered an independent risk factor for the development of AKI [1, 2]. The association between trauma severity and kidney damage could be explained by primary anatomic mechanisms, such as direct renal trauma and rhabdomyolysis secondary to crush injuries, and secondary physiologic effects, such as inadequate renal perfusion and altered inflammatory cytokines [32, 33]. Despite trauma severity being associated with AKI, the type of trauma was not. Approximately 75% of the overall patient population and AKI cases had experienced blunt trauma, limiting the comparisons between AKI and different trauma types. In people, blunt trauma is also generally the most commonly encountered trauma type (78.9% of cases in one study) and is associated with AKI in some [1, 34], but not other, studies [23].

Survivors of azotemic AKI in this study had shorter hospitalizations than non‐AKI cases, which is in contrast to human studies whereby trauma‐associated AKI is associated with prolonged hospitalization [35] or ICU hospitalization [23], but similar to other studies in which a difference was seen in ICU hospitalizations but not overall hospitalization length [21]. Although this was an unexpected finding, it is interesting to note that the median values of hospitalization for AKI and non‐AKI cases were similar (3.5 days and 4 days, respectively), which may reflect the difference in number of survivors in the AKI group (20 compared with 303 in the non‐AKI group), leading to a type II error. Future studies should focus on hospitalized days in the ICU and the effect of hospitalization on HAAKI.

AKI cases in this study had a survival of 78.6%, which is similar to a study in people that indicated 77.5% of AKI cases survive after trauma [35]. The small number of cases with azotemic AKI across each AKI grade makes it impossible to determine the effect of grade on survival. Although a small number of patients developed HAAKI, these cases experienced poor survival (42.9%), with 75% of deceased cases suffering cardiac arrest in hospital and 25% being euthanized for mixed reasons, making this an important area for future investigation and prognostication.

The relative hyperglycemia in AKI cases compared with non‐AKI cases is comparable to human data where multiple mechanisms have been proposed for dysglycemia in trauma, which may increase the risk of AKI [36]. Given the multiple factors influencing blood glucose concentrations in this population and the significant overlap of values between groups, clinical significance would be better assessed in a prospective study. No significance could be tested for in other groups due to low case numbers. In human trauma patients, the risk of developing AKI after undergoing surgical procedures and receiving blood products is well established [1, 11, 37, 38]. The requirement for a blood transfusion may be associated with AKI as it may be correlated with hemodynamic instability, injury severity, and antigen exposure [11, 39]. AKI is a recognized complication in 6% of veterinary patients undergoing mechanical ventilation [40] and human trauma patients on mechanical ventilation [41]. Future studies should explore these factors further in veterinary trauma.

Potentially nephrotoxic drugs were used in 25% (7/28) of azotemic AKI cases, with six of seven (85.7%) cases receiving meloxicam. Interestingly, three of seven (42.9%) HAAKI cases developed azotemia after undergoing computed tomography with contrast medium administration. There is debate in the literature regarding the prevalence of contrast‐associated AKI in people and veterinary species, with a recent veterinary study highlighting a temporal association between contrast administration and renal injury; however, causation could not be established [42, 43]. Due to missing equivalent non‐AKI data and a small AKI dataset, no conclusions can be made regarding the relationship between the use of these drugs and AKI, and further investigation is warranted.

### 4.1 Limitations and Future Directions

Due to the study's retrospective nature, a large number of patients with incomplete data were excluded, which may have resulted in an underestimation of the true prevalence of AKI secondary to trauma, particularly those in fluid‐responsive and HAAKI groups. Failure to detect a difference in survival between the azotemic AKI and non‐AKI cases may have been the result of a type II error. As the majority of cases were referred from other practices, the results of the current study may not reflect the overall prevalence of AKI after trauma in first opinion practice. It was not possible to determine the time from trauma to presentation and creatinine measurement due to inconsistencies in data recording. As such, attributing trauma as the cause of AKI is difficult when adjustment for other confounding variables (e.g., treatment with certain medications and fluids) could not be done. These cases were from a single trauma center in the United Kingdom, which may affect the generalizability of the data to other UK or international hospitals.

We defined AKI based on blood creatinine concentration, for which only 17 of 28 (60.7%) cases with AKI at presentation had serial values available to review; only one of these cases had UOP measured. UOP is key to detecting early grades of AKI and determining fluid responsiveness, without which lower grades of AKI cases may have been overlooked. Cases with preexisting kidney disease were excluded based on reports in the medical history; however, incomplete data alongside the prevalence of CKD in the general feline population make it possible that some AKI cases were unidentified acute‐on‐chronic kidney injuries. Future prospective studies assessing UOP and measuring urinary biomarkers are warranted to better assess the prevalence of AKI in small animal trauma patients.

Patients that were nonazotemic on presentation and had a documented increase in blood creatinine concentration ≥ 26.4 µmol/L (≥ 0.3 mg/dL) during hospitalization were considered to have HAAKI, which is in line with previous veterinary literature [9, 44]. Although there are inconsistencies in the human literature, HAAKI is often defined as the development of AKI any time after 48 h of hospitalization [45]. Including patients that developed AKI within 48 h may have resulted in the misclassification of community‐acquired AKI as HAAKI. Interestingly, of the cases that developed AKI after 48 h, two had their second blood sample at 72 h and one had its next blood sample at 9 days, making it difficult to determine when exactly azotemia developed. Further studies of HAAKI, as defined in the human literature, are needed to fully determine the true incidence of HAAKI. Analyzing the potential risk factors for development of AKI was hindered by the retrospective nature of this study, in particular making it difficult to assess fluid types, rates, and drug doses. Determination of true risk was also limited by the failure to screen the non‐AKI population for exposure to these risk factors.

Despite these limitations, this is the first study to specifically assess the prevalence of AKI in veterinary trauma patients, documenting azotemic AKI on admission after trauma in 13.8% of cats and 2.3% of dogs, of which 32.1% were volume responsive. Seven of 105 nonazotemic patients with serial blood work available (6.67%) developed HAAKI. Being a cat and having a higher ATT score may increase the risk of azotemic AKI on presentation. A prospective study documenting these variables alongside procedures and drug doses in both AKI and non‐AKI is warranted.

## Author Contributions

**Emily Jane Stacey**: Conceptualization; data curation; formal analysis; investigation; methodology; project administration. **Stefano Cortellini**: Conceptualization; data curation; formal analysis; investigation; methodology; project administration. **Laura Pearl Cole**: Conceptualization; formal analysis; investigation; methodology; project administration.

## Conflicts of Interest

The authors declare no conflicts of interest.

## Endnotes

a ABL800 FLEX, Radiometer Medical ApS, Copenhagen, Denmark to measure venous blood gas and creatinine.

b Abbott i‐STAT 1 analyser, Abbott, IL 60064, USA. Chem8 cartridge to measure creatinine.

c GraphPad Prism 9.3.1 (350).
